# Draft genome sequence of the highly copper-tolerant *Methylobacterium radiotolerans* MLP1 isolated from the rhizosphere of grasses adjacent to mine tailings

**DOI:** 10.1128/MRA.00361-23

**Published:** 2023-08-28

**Authors:** Araceli Dávalos, Luis Fernando Lozano-Aguirre Beltrán, Alejandro García-de los Santos

**Affiliations:** 1 Centro de Ciencias Genómicas, UNAM, Cuernavaca, Morelos, Mexico; University of Maryland School of Medicine, Baltimore, Maryland, USA

**Keywords:** *Methylobacterium radiotolerans* genome, copper homeostasis, high copper-tolerant bacteria, mine tailings, copper efflux resistome

## Abstract

We present the genome of a highly copper-tolerant pink-pigmented facultative methylotroph isolated from the rhizosphere of grasses growing close to mine tailings. Based on whole-genome taxonomic analyses, this isolate was named *Methylobacterium radiotolerans* MLP1. Studies are in progress to infer its genome-based copper resistome.

## ANNOUNCEMENT

To understand how is organized the Cu-efflux resistome in bacteria thriving in Cu-rich environments, soil suspension from the rhizosphere of grasses sampled in the inactive mine “La Prieta” (Hidalgo del Parral, Chihuahua, México, 26° 56´ 27´´N, 105° 39´ 32´´W) (1) was screened for the presence of highly copper-tolerant bacteria by dilution and spread plate technique (2). After 72 h of incubation at 30°C, the most copper-tolerant isolate showed full growth on plates of succinate-ammonium mineral medium (3) supplemented with 4.0 mM CuCl_2_ and total growth inhibition in 4.5 mM CuCl_2_ (MIC), which falls into the range of tolerance reported for other highly Cu-tolerant species (4, 5).

Its pink-pigmented phenotype and the ability to use methanol as the sole carbon source resembled pink-pigmented facultative methylotrophic bacteria (PPFM). This strain was named PPFM-MLP1. We report its genome sequence and taxonomic position.

The genomic DNA was extracted from a 5-mL bacterial culture, grown overnight in peptone-yeast broth, using the Bacterial Genomic DNA Isolation Kit (Norgen Biotec #17900). Sequencing was performed at UUSMB-UNAM with an Illumina Genome Analyzer II using NextSeq 500 platform dual-indexed paired-end reads. Two libraries were built from 3 µg of DNA by using Nextera XT DNA Library Preparation Kit. The sequencing yield resulted in 8,045,364 paired-end reads. Default parameters were used for all software unless otherwise specified. The raw data were quality filtered with FastQC software v0.11.9 (6) and Trim Galore software v0.6.4 with --clip_R1 and --clip_R2 value 15 (7). The genome was assembled using SPAdes v3.13.1 with kmer values of 21, 33, 55, 77, 99, 111, and 127 (8). A total of 104 contigs (*N*_50_ = 129,509) with 177× coverage were used for mapping with nucleotide MUMmer system v3.1 (9). Genome annotation was performed by NCBI PGAP v6.3 (10). The assembled draft genome had a total length of 6,717,541 bp, 100% completeness, with a G + C content of 71.4%, 6,331 CDS (6,224 with coding regions), 50 tRNA genes, and 1 complete rRNA gene.

**Fig 1:**
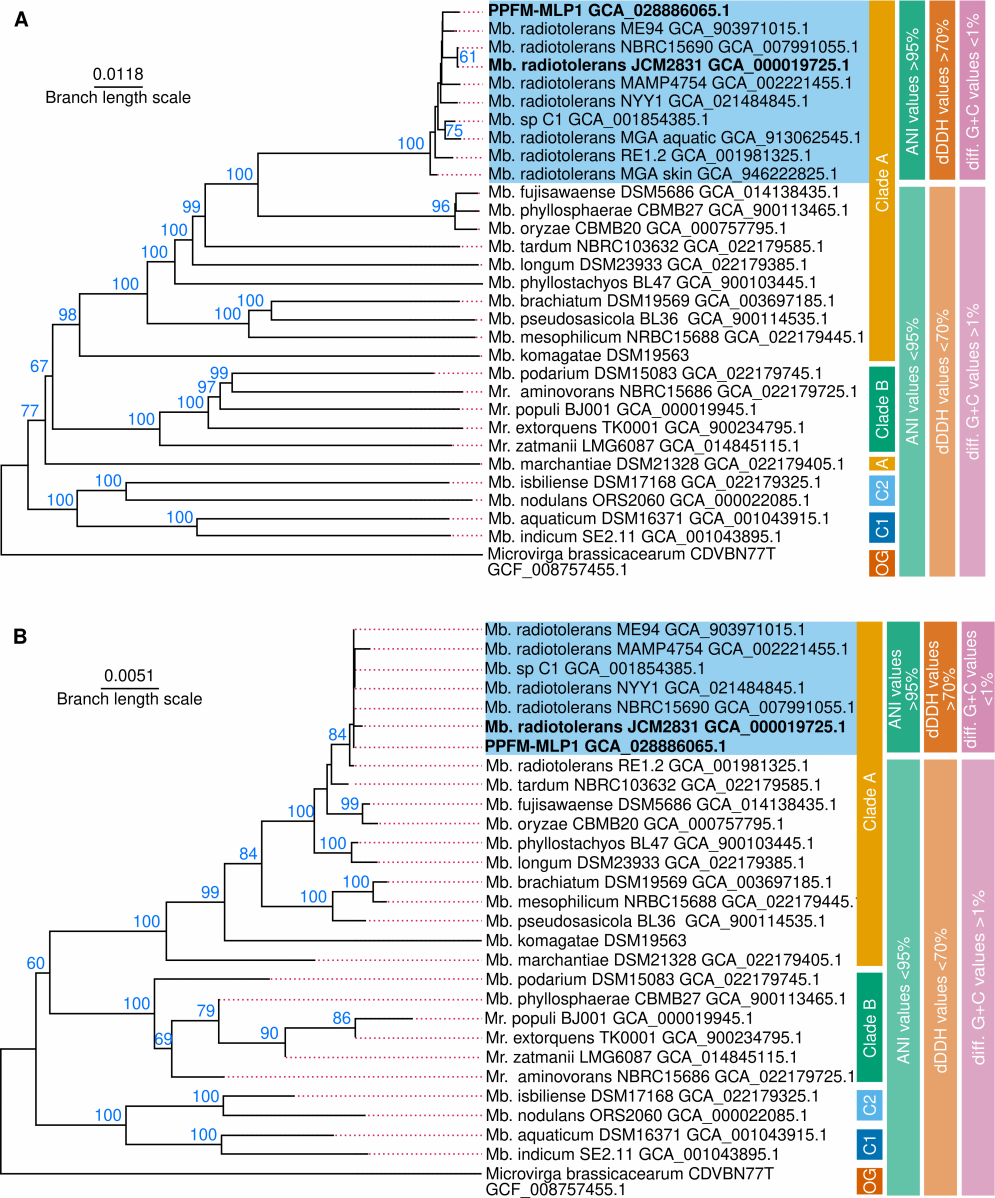
The PPFM-MLP1 is closely related to *M. radiotolerans* species. The figure integrates the results of four different GBTA used to delineate the taxonomic position of PPFM-MLP1 as part of *M. radiotolerans* lineage. The data set analyzed consisted of 30 genome sequences belonging to the four different clades reported for the phylogeny of the genera *Methylobacterium* (*Mb*) and *Methylorubrum* (*Mr*.) (13). The genus *Microvirga* represented by *Microvirga brassicacearum*^T^ was used as an outgroup (OG). The name of each strain and its respective GeneBank assembly accession numbers are indicated. The TYGS tree was inferred with FASTME 2.1.6.1 (14) from GBDP distances calculated from 30 genome sequences (**A**) or 28 16S rDNA sequences (**B**). The branch lengths are scaled in terms of GBDP formula *d_5_*. The numbers above the branches are GBDP pseudo-bootstrap values >60% from 100 replications. The tree was visualized with PhyD3 (15). ANI values were estimated with an ANI calculator using the OrthoANIu algorithm (16). The dDDH values were predicted with the dDDH calculator implemented in the TYGS server using the formula *d_4_* as recommended in reference (17). The G + C differences were calculated by the TYGS server. The cutoff values to define species boundaries are explained in the text.

The genome of PPFM-MLP1 was uploaded to the Type (Strain) Genome Server (TYGS) v 2022 to perform a genome-based taxonomic analysis (GBTA) based on Genome BLAST Distances Phylogeny (GBDP) using default settings (11). The comparison of query genome against type strains genomes in the TYGS database via the MASH algorithm (12) predicted that *Methylobacterium radiotolerans* JCM 2831^T^ was the type strain closest to PPFM-MLP1. A second GBTA was performed at the TYGS server adding eight genomes from *M. radiotolerans* strains reported in NCBI but not contained in the TYGS database. The phylogeny inferred from inter-genome distances and 16S rDNA sequences indicated the PPFM-MLP1 is part of the *M. radiotolerans* lineage (Fig. 1). The genomes of this cluster shared whole-genome average nucleotide identity (ANI) values >95%, digital DNA-DNA hybridizations (dDDH) values >70%, and G + C differences <1%, indicating that these genomes belong to the same species (see more details in Fig. 1). This isolate will be named *M. radiotolerans* MLP1.

## Data Availability

The whole-genome shotgun project was deposited in GenBank under the accession number JAPTHG010000000 (BioSample accession number SAMN32095585 and BioProject accession number PRJNA909792). The raw sequence reads have been deposited in the Sequence Read Archive (SRA) under the accession number SRR22690093. The GenBank assembly link is ASM2888606v1.
